# Exploratory randomized controlled trial evaluating the impact of a waiting list control design

**DOI:** 10.1186/1471-2288-13-150

**Published:** 2013-12-06

**Authors:** John A Cunningham, Kypros Kypri, Jim McCambridge

**Affiliations:** 1 Centre for Mental Health Research, the Australian National University, Canberra, Australia; 2 Centre for Addiction and Mental Health, Toronto, Canada; 3 Centre for Clinical Epidemiology and Biostatistics, School of Medicine and Public Health, University of Newcastle, Callaghan, NSW, Australia; 4 Faculty of Public Health and Policy, London School of Hygiene and Tropical Medicine, London, UK

**Keywords:** Randomized controlled trials, Research methods, Waiting list control design, Alternate explanation, Alcohol, Brief intervention

## Abstract

**Background:**

Employing waiting list control designs in psychological and behavioral intervention research may artificially inflate intervention effect estimates. This exploratory randomized controlled trial tested this proposition in a study employing a brief intervention for problem drinkers, one domain of research in which waiting list control designs are used.

**Methods:**

All participants (N = 185) were provided with brief personalized feedback intervention materials after being randomly allocated either to be told that they were in the intervention condition and that this was the intervention or to be told that they were in the waiting list control condition and that they would receive access to the intervention in four weeks with this information provided in the meantime.

**Results:**

A total of 157 participants (85%) were followed-up after 4 weeks. Between-group differences were found in one of four outcomes (proportion within safe drinking guidelines). An interaction was identified between experimental manipulation and stage of change at study entry such that participant change was arrested among those more ready to change and told they were on the waiting list.

**Conclusions:**

Trials with waiting list control conditions may overestimate treatment effects, though the extent of any such bias appears likely to vary between study populations. Arguably they should only be used where this threat to valid inference has been carefully assessed.

## Background

There has been growing concern over the use of waiting list control designs in psychological and behavioral intervention research [1-8]. While there are ethical advantages to a waiting list design because it allows for the provision of care (if delayed) to research participants who are seeking help, whilst permitting a non-intervention evaluation, it has been noted that such designs may overestimate intervention effects [1-7]. This is because participants assigned to a waiting list control condition appear to improve less (or not at all) than would be expected for people who are concerned about their behavior and who are taking steps to change. In a discussion of this possibility, Miller and Rollnick point out that examination of patterns of change in participants assigned to waiting list control conditions may indicate that they perceive they are expected to ‘wait’ to change until receiving the intervention and compliantly do so [9]. This contrasts with studies not employing waiting list designs in which control group participants tend to improve [10-12].

There has been some research on this topic, usually framed in the context of expectancies or demand characteristics, though there has been little dedicated study of the latter outside laboratory settings [13]. Previous studies have included a range of waiting list designs, some in which the participant is told that they will have to wait to receive treatment and others where a period of monitoring is described as a necessary baseline [14,15]. For the rare interventions where it is impossible for the participant to know whether they are receiving an intervention (e.g., distance healing), research has also been conducted where the presence or absence of the intervention is crossed with the participant being told that they are, or are not, receiving the intervention in order to estimate the impact of expectancies [16]. Other factorial designs have also been used to evaluate unintended impacts of the research process [17].

Brief interventions for problem (i.e. hazardous or harmful) drinking is one area of research utilizing waiting list control designs [18,19]. Given evidence that problem drinking is often resolved without treatment [10,20], the use of a waiting list control design may be unethical if it has the effects ascribed to it. While other aspects of the research process have been evaluated in this field [21], there has been no experimental study of the effects of waiting list control conditions on participant drinking, i.e., of reactivity within this research design. In this exploratory randomized controlled trial we sought to develop a method for testing two hypotheses: Hypothesis 1: When given the same intervention material, people told that they are in the waiting list control condition will report heavier drinking at follow-up than people told that they are in the intervention condition (a main effect); Hypothesis 2: Participants who are more ready to change their drinking will report heavier drinking at follow-up compared to participants who are less ready to change (an interaction effect).

## Methods

This study employed a ‘no difference’ trial paradigm in which all participants are given access to the same intervention while other aspects of the research process are experimentally manipulated [22]. Potential participants were recruited through Toronto newspaper advertisements inviting people ‘concerned about their drinking’ to help with the evaluation of self-directed interventions. The newspaper advertisement also mentioned that compensation would be provided and that the study was not a treatment program. Respondents telephoned study staff and were mailed out a consent form and baseline assessment questionnaire. Participants who returned the consent form and baseline questionnaire were randomized to be told that they were either in the: a) intervention condition and sent feedback generated from a known effective intervention, Check Your Drinking (CYD) website [23-26]; or b) waiting list condition and would be sent details of the intervention in 4 weeks and provided with information about their drinking in the meantime (which was identical to the feedback generated using the CYD website). See below for the exact content of this experimental manipulation. Follow-up was conducted four weeks after baseline and participants received $20 for returning the follow-up questionnaire. Both baseline and follow-up assessments were conducted by postal questionnaire. After the follow-up questionnaire was returned, all participants were provided access to the Alcohol Help Centre (AHC) online program and participants in the ‘waiting list control condition’ were told that this was the intervention [27]. Thus, the only difference between the two groups was that those in one condition were told that they were receiving an intervention and those in the other were told that they had to wait four weeks before getting access to the intervention. The follow-up length of four weeks was partly chosen to replicate a published trial of a similar web-based intervention for problem drinkers which used a waiting list control design [18].

### Text used as experimental manipulation

**Text used in intervention condition:** “You are in the intervention condition of this study. We have developed a personalized feedback intervention for people concerned about their drinking. We generated a Final Report for you from this intervention and it is included with this letter.”

**Text used in waiting list condition:** “You are in the waiting list condition of this study. You will need to wait for 4 weeks until we can send you the intervention materials. In the meantime, we have generated some personalized information about your drinking and it is included with this letter.”

### Ethics

After providing a description of the study to the subjects, written informed consent was obtained. This consent procedure and the conduct of the study were approved by the standing ethics review committee of the Centre for Addiction and Mental Health.

### Baseline and outcome variables and analysis plan

The outcome variables were: number of drinks in a typical recent week, largest number of drinks on one occasion, and the AUDIT-C [28], the consumption subscale of the Alcohol Use Disorders Identification Test AUDIT, [29,30], which includes three items (frequency of drinking, number of drinks per drinking day, and frequency of five of more drinks on one occasion) and total scores range from 0 to 12. The final outcome measure was unplanned prior to the completion of the study and comprised the proportion of participants drinking within the Canadian safe drinking guideline (for males, no more than 15 drinks per week and three drinks per drinking day; for females, no more than 10 drinks per week and two drinks per drinking day) [31]. The questionnaire contained a graphic describing a standard drink which in Canada contains 13.6 grams of ethanol [31]. These variables were assessed at baseline and at follow-up. In addition, at baseline, participants provided information on their demographic characteristics, and completed the other items of the full AUDIT and the Readiness to Change (RTC) questionnaire [32,33].

Continuous outcome variables were examined for outliers and Winsorized to normalize the distribution by replacing values more than three standard deviations from the mean with the next highest value. Analyses for the continuous variables were conducted using stepwise linear regression in which the baseline value of each outcome variable was entered in Step 1. In Step 2, participant experimental condition was entered. In addition, the Action subscale of the RTC was entered as a main effect continuous variable. Finally, in Step 3, an interaction term was added. The categorical variable, proportion of participants drinking within safe drinking guidelines, was compared between experimental conditions using Fisher’s exact test. Note that an earlier version of this analysis was conducted using logistic regression in order to allow the inclusion of the Action subscale as one of the predictors (including a main effect and an interaction term with experimental condition). However, as there was no significant (*p* > .05) main effect or interaction effect of the Action subscale, and because the inclusion of the Action subscale did not substantively influence the main effect of experimental condition observed, the simpler Fischer’s exact test is presented in this paper. Data missing at follow-up were not replaced to mimic the treatment of missing data employed in studies evaluating brief interventions of this type with waiting list control designs [18] and because attrition was not judged likely to be problematic^a^.

### Study participants

A total of 191 participants responded to the newspaper advertisements and returned a signed consent form and baseline questionnaire. Of these, 185 were hazardous or harmful drinkers (as defined by an AUDIT score of 8 or more) and were included in this study. Bivariate comparisons were made on demographic and baseline drinking characteristics between experimental condition and there were no significant differences (*p* > .05). The mean age of the 185 participants was 47.3 (SD 11.4), 70.3% were male, 55.1% had some post-secondary education, 31.4% were married or living in common law relationships, 52.4% were full or part-time employed, and 48.1% reported a family income of less than $30,000 per year. Baseline levels of problem drinking were quite severe for a community-recruited sample with a mean AUDIT score of 24.1 (SD 7.0; a score of 20 or more on the AUDIT is indicative of possible alcohol dependence). Participants reported typically consuming an average of 35.3 (SD 21.4) drinks per week, and the mean highest number of drinks consumed on one occasion in the last year was 14.7 (SD 7.5). Participants’ baseline AUDIT-C mean score was 9.0 (SD 2.0).

## Results

**Figure 1:**
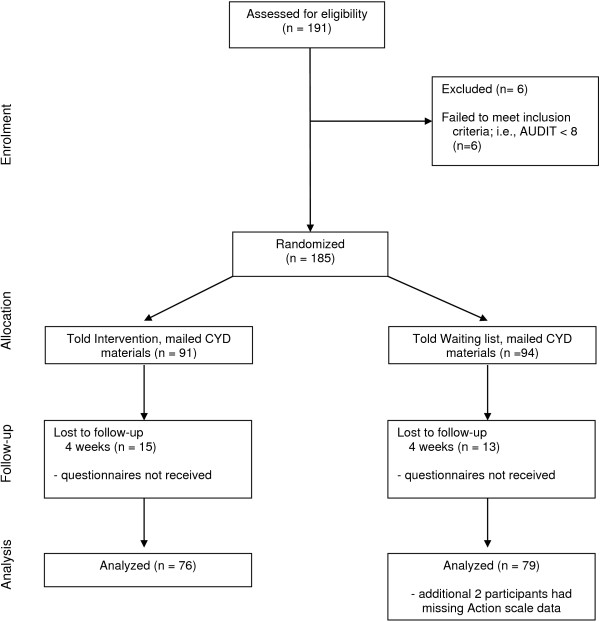
CONSORT flowchart.

Follow-up rates were satisfactory with 157 (85%) returning their four-week survey. An additional two participants did not complete the items for the Action subscale at baseline, leaving 155 participants available for analysis. Figure 1 provides displays an overview of the recruitment and follow-up rates of the trial.

There were no significant differences in follow-up rates between experimental condition (Told intervention condition = 83.5%; Told waiting list = 86.2%; *p* = .68). Table 1 displays the data for the three regression analyses. In order to facilitate interpretation of these analyses, Table 2 displays the means (SD) of the three continuous variables at baseline and follow-up, separated by experimental condition and a median split on participants’ baseline Action subscale scores. For the outcome variable, typical drinks per week, there was a significant interaction between experimental condition and the Action subscale (*p* = .05). Inspection of the estimated marginal means on Table 2 revealed that for participants who rated themselves as low on the Action subscale, condition allocation (waiting list or intervention) had little or no impact on their drinking. However, participants who scored themselves above the median on the Action subscale and who were in the waiting list condition reported drinking about 6 drinks per week more than their counterparts in the intervention condition. A similar pattern of results was observed for the largest number of drinks consumed on one occasion (*p* < .01). There were no significant experimental condition or interaction effects (*p* > .05) for AUDIT-C scores.

**Table 1:** Relationship of four-week follow-up drinking with intervention condition and level of action for change intent after controlling for baseline drinking

	**Drinks per week**		**Highest number**		**AUDIT-C**^**a**^	
**Predictor**	∆R^2^	β (95% C.I)	∆R^2^	β (95% C.I)	∆R^2^	β (95% C.I)
**STEP 1**	.32***		.45***		.19***	
Baseline drinking		0.46 (0.35 - 0.56)***		0.50 (0.41 - 0.59)***		0.59 (0.39 - 0.79)***
**STEP 2**	.05**		.01		.06**	
Condition^b^		1.51 (-2.71 – 5.73)		0.45 (-0.84 – 1.74)		0.27 (-0.50 – 1.03)
Action subscale		-0.87 (-1.41 - -0.32)**		-0.12 (-0.29 – 0.44)		-1.65 (-0.26 - -0.06)***
**STEP 3**	.02*		.03**		.01	
Condition*Action		1.08 (0.00 - 2.16)*		0.48 (0.16 - 0.81)**		0.14 (-0.06 – 0.34)

*Note:* *p ≤ .05. **p ≤ .01. ***p ≤ .001.

^a^AUDIT-C is a composite measure that consists of respondents scores on frequency of drinking, drinks per drinking day, and frequency of five or more drinks on one occasion.

^b^Condition: 0 = told in intervention condition; 1 = told in waiting list condition.

**Table 2:** **Mean ( *SD *) drinking variables at baseline and four-week follow-up by study condition (told in intervention versus told on waiting list) and median split on Action stage variable (N = 155)**^a^

	**Low action score**		**High action score**	
	**Told intervention (n = 36)**	**Told waiting list (n = 46)**	**Told intervention (n = 40)**	**Told waiting list (n = 33)**
**Typical weekly drinking [Mean (SD)]**				
Baseline	32.8 (17.7)	33.4 (20.2)	32.6 (16.8)	35.5 (25.3)
4-week	25.0 (17.7)	24.2 (17.7)	14.5 (9.8)	21.4 (15.5)
**Highest number on one occasion [Mean (SD)]**				
Baseline	12.9 (5.7)	14.1 (8.4)	15.3 (5.8)	14.1 (8.4)
4-week	8.6 (4.5)	8.5 (5.4)	7.6 (5.3)	8.9 (6.3)
**Audit-C score**^**b **^**[Mean (SD)]**				
Baseline	8.9 (1.7)	8.8 (2.3)	9.2 (1.8)	9.0 (2.0)
4-week	6.9 (2.5)	7.1 (2.7)	5.7 (3.0)	6.1 (2.4)

^a^Analyses employed Action scale as a continuous measure (see Table 1). Median split values on this table are provided for descriptive purposes.

^b^AUDIT-C is a composite measure that consists of respondents scores on frequency of drinking, drinks per drinking day, and frequency of five or more drinks on one occasion. Scores range from 0–12.

### Proportion drinking within safe drinking guidelines

Despite scoring 8 or more on the AUDIT, 4.3% of participants [(8/185; or 4.5% (7/157) of participants followed-up], were drinking within safe drinking guidelines at the time of the baseline assessment. At follow-up, 20.4% (32/157) were drinking within safe drinking guidelines. There was a main effect of study condition, both on the proportion of participants drinking within safe drinking guidelines at follow-up (Fisher’s Exact test, *p* = .03; Told intervention condition = 27.6%; Told waiting list = 13.6%) and on the increase in proportion of those who did not drink within safe drinking guidelines at baseline but did so at follow-up (Fisher’s Exact test, *p* = .009; Told intervention condition = 25.0%; Told waiting list = 8.6%).

## Discussion

This exploratory trial developed a research design to examine the effects of being in a waiting list control condition in psychological and behavioural intervention research. The intention behind this study design was to achieve a contrast between the effect of intervention deliberately confounded with expectancy (as is usually delivered and evaluated), versus the effect of intervention without expectancy, thereby estimating the expectancy effect as the difference between the two. Specifically, the design allows for a test of the impact of being told that the participant is in the waiting list control condition because all participants are provided with the same intervention materials (the personalized feedback report generated from the CYD online program). This design is equivalent to the subtractive expectancy placebo proposed by Suedfeld [34] where all participants are given the intervention but a randomized half is led to expect that it is inert with respect to the problem being treated. The advantage of the personalized feedback material for this particular study was that it was brief and could credibly be described as merely information to those in the waiting list control condition, while at the same time being a plausible intervention for those in the other study condition. As such, the feedback has intrinsic placebo properties and this intervention is not different in this respect from other interventions.

Mixed findings were obtained in relation to Hypothesis 1, only in the sense that while a lower proportion of participants in the control condition reported drinking within recommended guidelines at follow-up than their intervention group counterparts (an originally unplanned analysis), there were no between-group differences in the number of drinks per week, highest number of drinks on one occasion, and the heaviness of drinking measured with the AUDIT-C (the three planned outcome measures). The observed differences were in the anticipated direction and are generally coherent with an expectation that they would be statistically significant in a larger sample. Our test of Hypothesis 2 strengthens this possibility as it demonstrated heavier drinking among waiting-list control participants who were more ready to change compared to their counterparts receiving the “intervention.” This finding is consistent with the overarching hypothesised mechanism of effect, that waiting list allocation interrupts efforts at change, and also points to the importance of consideration of readiness for, or activities towards, change in this regard.

While it would be unjustified to conclude that a waiting list effect exists on the basis of statistically significant main effects from one of the four outcomes, the interaction effects observed here are provocative. For two of the continuous outcome variables, largest number of drinks on one occasion, and number of drinks in a typical week, there were interactions between experimental condition and readiness to change (started to do something about their drinking on entering the trial). It should be noted that these analyses were conducted in two different ways – the first using the categorical stages of change designation calculated using the full RTC scale [32] and the second employing just the action subscale. This repetition of analyses is justified given the exploratory nature of this trial, though the lack of an *a priori* constructed data analysis plan is acknowledged. Also, the finding that only the action subscale was predictive of outcome aligns with findings from previous research on Stages of Change albeit using a different measure of the construct [35].

Despite the strengths in the employed research design, one limitation may also be implicit in it. An additive model is assumed in that both groups are intended to actually read the feedback report [36]. Any differences between groups in doing so, will serve to inflate apparent waiting list effects entirely due to expectancies. Specifically, if being told that being in the intervention condition makes one more likely to read, or to think about, the intervention material, then the nature of the effect involves an interaction and is thus more complex [36]. This means that the effects observed here are contingent upon this specific feature of this study. The exploratory nature of this study also imposes various limits to inference. A power calculation was not undertaken *a priori*, making both the attainment of statistical significance here less important, and relatedly, clear and unambiguous interpretation of study findings more challenging. Effect estimates for future larger replication studies are now available in this study [37]. Another limitation was that there was no way to distinguish between those who were responding to the newspaper advertisements because they were looking for help regarding their drinking and those more motivated by the $20 for participating in the trial. To the extent that the financial incentive motivated participation it is likely that the observed differences under-represent the size of the true effects, particularly in light of the observed readiness to change findings. Alternatively, if the newspaper recruitment resulted in a sample that was highly motivated to change (e.g., in comparison to a proactively recruited sample) then the results of this trial could overestimate the impact of a waiting list design in such a population. The latter possibility is highly unlikely in treatment studies for help seekers, but should be borne in mind for brief intervention trials based on opportunistic recruitment in healthcare settings.

Future directions for this research include examining whether a waiting list control manipulation has more impact in particular research settings and with specific populations. For example, in situations where the manipulation is delivered face-to-face, more reactivity relative to the waiting list control condition may result. There is obvious value in examining the mechanisms behind the hypothesised negative impact of the waiting list control condition. This is particularly true for study populations of confirmed help-seekers. Qualitative interviews could also be used to investigate negative reactions of participants assigned to waiting list or other types of control conditions, particularly among those with clear preferences. Separating true expectancy effects associated with compliance with demand characteristics implicit in waiting list study conditions from participants becoming irritated or disconsolate over not getting the help they hoped to receive and reduce their own efforts to drink less (termed *resentful demoralization*[38]) will be a further challenge to address when this field of investigation is more developed.

## Conclusions

The results of this exploratory study give further weight to the generally increasing levels of scrutiny recently given to control conditions, and for the interpretation of findings from trials employing wait-list control designs in particular. Further, these results point to the need for caution regarding the ethics of assigning participants actively ready to change to a waiting list control condition.

## Endnote

^a^At the suggestion of one of the reviewers, the primary analyses were re-conducted using an intention-to-treat approach (missing data at follow-up replaced with the respective baseline values). However, as the pattern of results was unchanged from that reported here, this alternative analysis was not reported in this paper.

## Competing interests

The authors have no competing interests to declare.

## Authors’ contributions

JAC, KK, and JM conceived of the study. JAC conducted the study and the analyses. JAC wrote the first draft of the paper. KK and JM revised subsequent drafts of the paper. All authors have read and approved the final version of the manuscript.

## Pre-publication history

The pre-publication history for this paper can be accessed here:

http://www.biomedcentral.com/1471-2288/13/150/prepub
